# Frailty and Trajectories of Blood Pressure Among Older Mexican Americans Over Time

**DOI:** 10.1093/geroni/igaa057.862

**Published:** 2020-12-16

**Authors:** Soham Al Snih, Martin Rodriguez, Lin-Na Chou, Kyriakos S Markides, Kenneth Ottenbacher

**Affiliations:** The University of Texas Medical Branch at Galveston, Galveston, Texas, United States

## Abstract

The objective of this study was to examine whether blood pressure (BP) trajectories differ by frailty status among older Mexican Americans. Data are from an 18-year prospective cohort study of 1,781 non-institutionalized Mexican American aged ≥ 67 years from the Hispanic Established Population for the Epidemiological Study of the Elderly (1995/96-2012/13). Frailty was defined as meeting three or more of the following: unintentional weight loss of >10 pounds, weakness, self-reported exhaustion, low physical activity, and slow walking speed. General linear mixed models were used to estimate trajectories of systolic and diastolic BP over an 18-year period as a function of frailty status. All variables were analyzed as time-dependent covariates except for gender and education. At baseline, 46.3% participants were non-frail, 44.8% were pre-frail, and 9.0% were frail; and the mean of systolic and diastolic BP was 136.9 mmHg (SD=18.6) and 77.3 mmHg (SD=12.2), respectively. Frail participants had greater systolic and diastolic BP decline over time than non-frail (estimate=-3.94, SE=0.88, p-value=<0.0001 and estimate=-1.32, SE=0.54, p-value=0.0138, respectively); and pre-frail participants had greater systolic BP decline than non-frail (estimate=-1.51, SE=0.54, p-value=0.0049), after controlling for all covariates. Those with high body mass index and hypertension with and without treatment had increased levels of systolic and diastolic BP over time. Older age, female gender, arthritis, diabetes, and stroke had decreased levels of diastolic BP over time. This study showed progressive decline in systolic and diastolic BP in frail compared to non-frail older Mexican Americans, which might have implications when treating frail older adults with hypertension.

